# A Data Mining-Based Analysis of Medication Rules in Treating Bone Marrow Suppression by Kidney-Tonifying Method

**DOI:** 10.1155/2019/1907848

**Published:** 2019-02-03

**Authors:** Xing You, Yinkun Xu, Jin Huang, Yaofeng Zhi, Fengzhen Wu, Fuping Ding, Jiaying Liang, Zhihui Cui, Jingyan Xu, Jin Zhang

**Affiliations:** ^1^School of Basic Medicine, Guangzhou University of Chinese Medicine, Guangzhou, Guangdong Province, 510006, China; ^2^The Research Center of Basic Integrative Medicine, Guangzhou University of Chinese Medicine, Guangzhou, Guangdong Province, 510006, China; ^3^School of Medical Information Engineering, Guangzhou University of Chinese Medicine, Guangzhou, Guangdong Province, 510006, China; ^4^School of Nursing, Guangzhou University of Chinese Medicine, Guangzhou, Guangdong Province, 510006, China

## Abstract

**Objective:**

To investigate the rule of kidney-tonifying method in Chinese medicine for the treatment of bone marrow suppression (BMS), in order to provide evidence and references for the clinical application of herbs and formulae.

**Design:**

Collecting and sorting the information about the treatment of BMS related to kidney-tonifying (Bushen) method in Chinese medicine literatures on databases including Chinese National Knowledge Infrastructure (CNKI), and Chinese Biomedical Literature Database (CBM), establishing a database of BMS treating formulae after radiotherapy and chemotherapy with traditional Chinese medicine (TCM) kidney-tonifying method, and finally applying the relevant theories and techniques of data mining to analyze the medication rules of it.

**Results:**

A total of 239 formulae and 202 herbs were included in this database, in which the herbs occurred 2,602 times in general. The high frequency herbs included Astragali Radix (Huangqi), Atractylodis Macrocephalae Rhizoma (Baizhu), and Ligustri Lucidi Fructus (Nvzhenzi). The main herb categories were deficiency-tonifying herbs, blood-activating herbs, dampness-draining diuretic herbs, heat-clearing herbs, and digestant herbs. Deficiency-tonifying herbs accounted for 64.60% of the total number. A total of 8 clustering formulae are summarized according to cluster analysis and 26 herb suits association rules are identified by Apriori algorithm.

**Conclusion:**

The treatment of BMS is mainly based on the method of invigorating the spleen and tonifying the kidney and liver to strengthen healthy qi, supplementing with blood-activating herbs, and dampness-draining diuretic herbs to eliminate pathogenic factors.

## 1. Introduction

BMS is one of the major side effect that is produced during the treatment of cancer patients with radiation, chemotherapy and drugs, which affect seriously patients' radio- and chemotherapical process and even led to treatment failure, which has become a major and difficult problem in clinical practice [1, 2]. According to its syndrome manifestation, BMS belongs to consumptive disease or blood deficiency problem in TCM, and the corresponding curative effects have been achieved by using the methods of invigorating kidney qi, strengthening and replenishing spleen qi, and activating blood and eliminating pathogenic factors [3, 4]. In particular, the kidney-reinforcing method based on the theoretical basis of traditional Chinese medicine, which is “kidney domains bone and produces marrow” and has been widely used due to its remarkable clinical effects, about which a large amount of clinical and experimental literatures are accumulated. Therefore, it is possible to apply data mining technology to analyze more effectively the mode of herbal prescription from the literatures, to explore the key herbs and common ones, and to discover the potential associations of them, which may benefit the diagnosis, treatment, and provision of BMS [5, 6].

## 2. Materials and Methods

### 2.1. Data Source and Normalization

By collecting and collating the literatures on the databases including CNKI, CBM, VIP database, and WANFANG database about the treatment of BMS after radiotherapy and chemotherapy in Chinese medicine and inputting them to the NoteExpress literature management software and eliminating the literatures which do not meet the requirements, such as reviews, thin sample or not representative cases, and proven reports and animal experiments, this study established a data information collection form and entered the filtered prescription information into it. When the data was collected and sorted, a total of 621 effective formulae were gained to build a database of Chinese medicine treatment of BMS. By inputting the keyword* kidney-tonifying*, 239 related formulae were retrieved, upon which a database of BMS formulae was established and was standardized and classified according to the standard name and Chinese medicine classification of* People's Republic of China Pharmacopoeia *(2015 Edition) [7] and* Chinese Medicine Dictionary* [8].

### 2.2. Data Processing and Analysis

A database of BMS treatment with kidney-tonifying formulae was established with the application of Excel 2013 and was converted into the format required by the data mining software. Programming and modelling the data with R language are done according to the data characteristics of Chinese medicine medication rules, within which the core herbs were clustered by Hierarchical clustering algorithm. For example, Vania M. Youroukova et al. analyzed the phenotype of severe bronchial asthma by using cluster analysis and obtained four clusters that provided reference for clinical treatment [9]. The Apriori algorithm in R language data mining software is used to analyze the association rules of core herbs, which is similar to Mateen Shaikh's applying association rule replacement test to test the relationship between genotype and phenotype, and deduced the genotype of candidate population [10].

## 3. Results

### 3.1. Descriptive Analysis Results

#### 3.1.1. Herb Frequency and Analysis

Among the 239 formulae included in the analysis, there were 202 herbs of Chinese medicine included, and their occurrence frequency was 2, 602 times. Of the highest frequency was the herb Astragali Radix (Huangqi), which occurs 184 times, with a frequency rate of 7.07%. The second was Atractylodis Macrocephalae Rhizoma (Baizhu), whose frequency was 129 times (4.96%); the Ligustri Lucidi Fructus (Nvzhenzi) ranked the third with 125 times and (4.80%). And the top-nine herbs, respectively, are Astragali Radix (Huangqi), Atractylodis Macrocephalae Rhizoma (Baizhu), Lucidi Fructus (Nvzhenzi), Angelicae Sinensis Radix(Danggui), Lycii Frucyus(Gouqizi), Codonopsis Radix (Dangshen), Poria(Fuling), Rehmanniae Radix Praepapata (Shudihuang), and Spatholobi Caulis (Jixueteng), whose frequencies all exceed 95 times, with their frequency rate higher than 3.65%, and their cumulative frequency reached 41.12%. Herbs with a frequency more than 18 times are shown in Table 1, their properties were mainly warm and mild, and the cumulative frequency of formulae was 66.83%. The tastes of the herbs were mainly sweet, bitter, and pungent. The frequency of herbs meridian tropism into the spleen, kidney, and liver meridians surpassed 30%, among which the frequency of the spleen meridian was of the highest, which was 47.58%. The details of herbs properties distribution are shown in Table 2, and the herbs flavors details are shown in Table 3, and herbs meridian tropism is shown in Table 4.

#### 3.1.2. Frequency and Analysis of Herb Categories

One hundred and eight high-frequency herbs with a frequency of 9 or more were categorized in this study. The details of herb categories and frequency are shown in Table 5, in which the top seven herb categories for treating BMS are deficiency-tonifying herbs, blood-activating herbs, dampness-draining herbs, heat-clearing herbs, digestant herbs, astringent herbs, and qi-regulating herbs, of which the cumulative frequency reached 80.46%, and deficiency-tonifying herbs occurred 1681 times which counts for 64.60% of the total number of uses and topped the list.

### 3.2. Cluster Analysis Results

**Figure 1:**
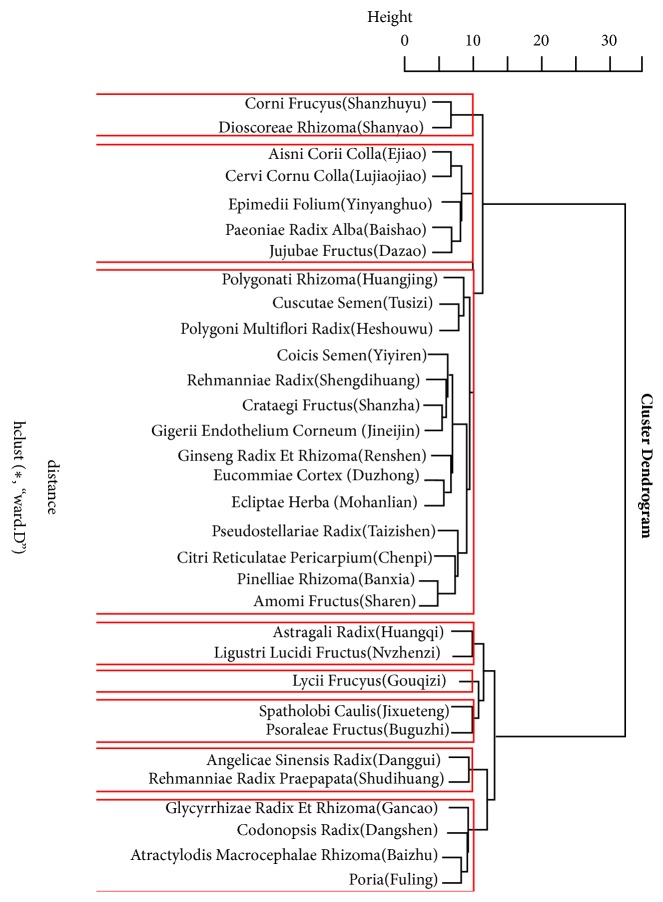
Cluster analysis tree diagram.

Thirty-eight core herbs mentioned above were clustered by Hierarchical clustering algorithm in R language and were divided into 4-10 classes by system clustering. Considering Chinese medicine theory, 8 categories are more appropriate in this study, and they were named as cluster one to eight subsequently. The clustering details are shown in Figure 1.

### 3.3. Results of Association Rules Analysis

**Figure 2:**
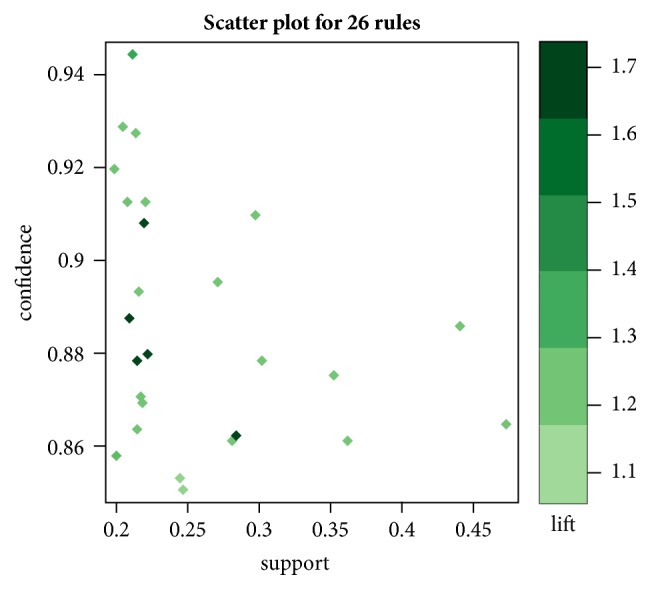
Association rule scatter plot.

**Figure 3:**
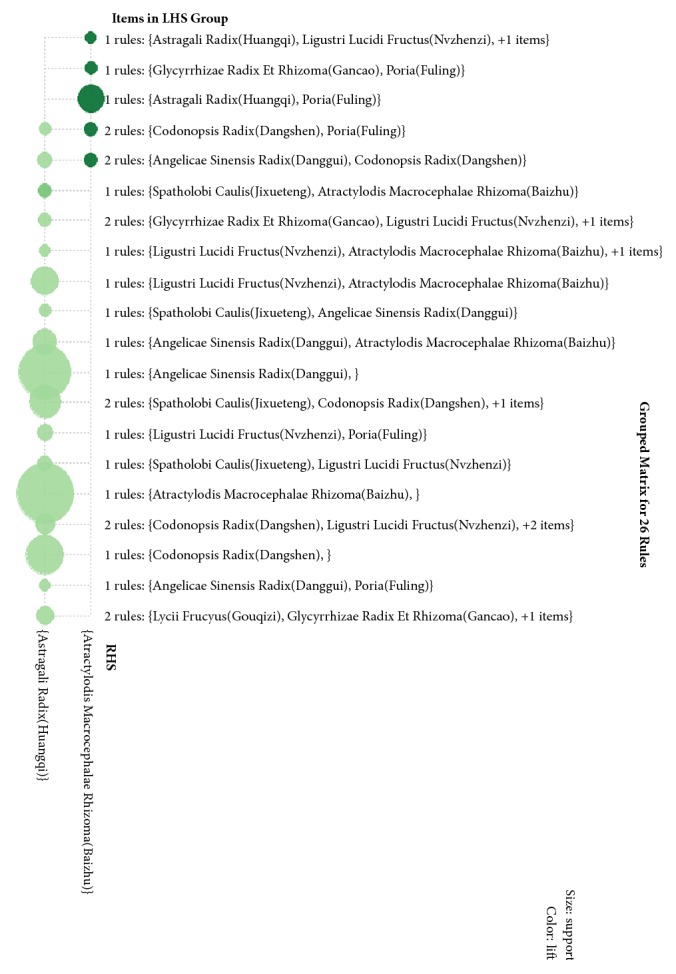
Association rule combination matrix.

**Figure 4:**
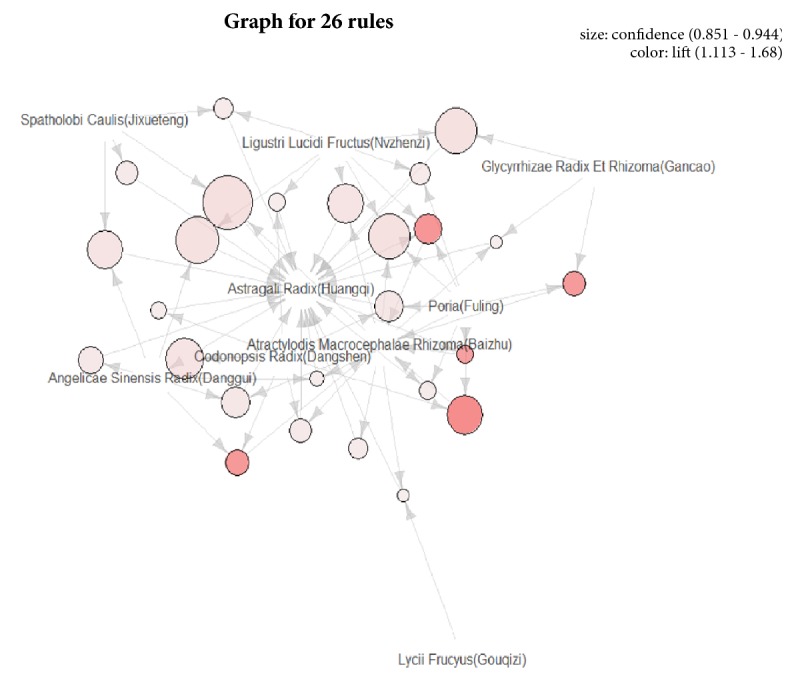
Association rule diagram.

In this study, the R language data mining software was applied for modeling, and the Apriori algorithm was used to analyze the association rules of the core herbs above. The parameters were set as support degree ≥20% and confidence level ≥85% [11], upon which a total of 26 herb pairs and suits association rules were obtained, and the details of the association rules are shown in Table 6. The correlation rules are shown in Figure 2 and the combination matrix is shown in Figure 3, and visualization is shown in Figure 4. In Table 6, {Atractylodis Macrocephalae Rhizoma(Baizhu)} =>{Astragali Radix(Huangqi)} is of the highest degree of support, which is 47.06%. {Codonopsis Radix(Dangshen), Poria(Fuling)}=>{Atractylodis Macrocephalae Rhizoma(Baizhu)}  {Atractylodis Macrocephalae Rhizoma(Baizhu), Spatholobi Caulis(Jixueteng)} => {Astragali Radix(Huangqi)} is of the highest confidence, which is 94.44% and {Codonopsis Radix(Dangshen), Poria(Fuling)} => {Atractylodis Macrocephalae Rhizoma(Baizhu)} is of the highest degree of promotion, which is 1.68.

## 4. Discussion

### 4.1. Strengthening the Spleen and Tonifying the Kidney and Supplementing the Liver and Kidney Are the Main Therapeutic Methods

(1) The method of strengthening the spleen and tonifying the kidney is the most common method for treating BMS in kidney-tonifying method. The herbs used in the tonifying kidney are mainly sweet, bitter, and pungent, rather than salty which is the corresponding flavor of the kidney in Chinese medicine theory and is different from the general idea that herbs salt in flavor are mainly used in kidney-reinforcing therapies and is more practical for clinical use [12]. The herbs enter spleen meridian topping the list, following the herbs to kidney and liver meridians. And all the association rules contain qi-invigorating herbs of the deficiency-tonifying category, whose main function is replenishing spleen and invigorating qi, which shows that tonifying spleen and kidney in kidney-tonifying method is most commonly used in treating BMS. Chinese medicine believes that the spleen and stomach are the postnatal basis of human body, in which ingesting food transforms it into essence to the body. The kidney is the congenital foundation which stores the essence and is in charge of yin and yang generation and transformation. The congenital and acquired essence mutually assist each other, thus tonifying the spleen and kidney can achieve an indirect effect of kidney-tonifying. Compared to simply tonifying kidney, the method to tonifying both spleen and kidney provides a flexible way in Chinese medicine clinical prescription formulation and compatibility, and herbs in the cluster group 4 and 8 are referred in the method according to the database. The cluster group 4 consisted of qi-invigorating and spleen meridian entry herb Astragali Radix (Huangqi) [13] and yin-nourishing and liver and kidney meridian entry herb Lucidi Fructus (Nvzhenzi) [14], with the effects of invigorating the spleen and kidney, tonifying qi and nourishing yin. The herbs in cluster group 8 are the main herbs in Sijunzi formula, a commonly used formula for clinical treatment of BMS in TCM. For example, Chen Jiaojiao et al. used the modified Sijunzi formula to treat BMS after lung cancer chemotherapy and achieved significant therapeutic effects [15]. (2) Tonifying the liver and kidney is another important treatment method for BMS in kidney-tonifying method. Both kidney and liver belong to the lower Jiao of human body, in which the kidney stores essence and liver stores blood, and the essence and blood mutually generate the counterpart; thus there is a saying in TCM that essence and blood are of the same source. Therefore, tonifying the liver and kidney can achieve the effect of tonifying the kidney directly compared to tonify the spleen and kidney. It can be seen from the clustering formulae that the formulae for tonifying liver and kidney can be divided into the general formulae for tonifying qi, blood, yin, and yang and the small formulae with less herbs and specific effects, from which we can see the two different perspectives of Chinese medicine in prescription of the kidney-tonifying method. One is applying the general formulae with various herbs of strong flavors to treat both the primary and secondary problems at one move, such as in cluster group 2 and cluster group 3 there are mainly deficiency-tonifying herbs which and tonify qi, blood, yin and yang. There are differences between the two. Cluster group 2 supplements without eliminating and is suitable for diseases of deficiency without pathogenic factor pattern, while in group 3, the deficiency-tonifying herbs are the basis and combined with herbs that eliminate pathogenic factors and promoting digestion and regulate qi to treat heat-toxin and phlegm-dampness and can be prescripted for patients whose symptom was defined as intermingled excess and deficiency. The second is based on the theory of yin and yang harmony. Kidney belongs to Shaoyin meridian, which is characterized as the governor of human body. It is believed that disease of this meridian could be treated by using less herbs in kinds and weight. For example, the cluster group 1 consisted of Corni Frucyus (Shanzhuyu) and Dioscoreae Rhizoma (Shanyao), with the functions of replenishing liver and kidney, nourishing essence, and bone marrow. There is only one herb Lycii Frucyus (Gouqizi) in cluster group 5, which is a common food in China, and the glutinous polysaccharide (LBP) contained in it has a therapeutic effect on BMS [16, 17]; thus the herb itself can be regarded as kidney-tonifying herb and food.

### 4.2. The Application of Eliminating Methods, Including Dampness-Draining Method and Blood-Activating and Stasis-Removing Method

The top nine frequent herbs are all deficiency-tonifying herbs apart from Poria (Fuling), the dampness-draining diuretic herb and Spatholobi Caulis (Jixueteng), the blood-activating herb. Although the two herbs are not classified as deficiency-tonifying herbs, they are often used in combination with deficiency-tonifying herbs to prevent the stagnation caused by deficiency-tonifying herbs. A former study shows that alfalfa polysaccharides and their derivatives have the effects of anticancer, anti-inflammatory, antioxidant, and antiviral activity biological activities and are good for health [18]. In in vitro and in vivo zebrafish embryo angiogenesis studies, Spatholobus suberectus extract can improve the cytotoxicity and angiogenesis of human umbilical vein endothelial cells induced by VRI (tyrosine kinase inhibitor II) [19]. In the treatment of BMS by kidney-tonifying formulae, apart from the deficiency tonifying herbs, the top herbs include blood-activating and stasis-removing herbs, dampness-draining herbs, digestant herbs, heat-clearing herbs, astringent herbs, and qi-regulating herbs. The herbs categories involved in the herb pairs and suits under association rules are deficiency-tonifying herbs, dampness-draining diuretic herbs and blood-activating, and stasis-removing herbs. Therefore, it can be concluded that, in the process of treating BMS after radiotherapy and chemotherapy, the main therapeutic methods are the kidney-tonifying method combined with dampness-draining and blood-activating method and add up with digestant, qi-regulating, and heat-clearing herbs. It can be clear that when tonifying healthy qi, kidney-tonifying method also combines with eliminating herbs to expel pathogenic factors, which conforms to relative clinical reports [20]. The reinforcing and reducing method in TCM are mutual benefiting and corresponds to the law of unity and opposition of yin and yang, it works to achieve the effect of tonifying and eliminating, and reflects the therapeutic principles of strengthening healthy qi and eliminating pathogenic factors.

## 5. Conclusions

According to the relevant techniques and theories of data mining and the analysis of medication rules of applying kidney-tonifying method in treating BMS after chemoradiotherapy, it is revealed that this kind of treatment is based on the method of strengthening spleen and tonifying kidney, replenishing liver and kidney and combined with invigorating healthy qi, and also applied methods include dampness-draining and blood-activating to eliminate pathogenic factors. With the help of data mining techniques and theories, this study has summarized the medication rules of kidney-tonifying method in treating BMS after radiotherapy and chemotherapy, which can facilitate TCM diagnosis, treatment, and experimental research, and would be helpful for BMS patients.

## Figures and Tables

**Table 1:** Herbs used over 40 times in prescriptions.

No.	Herb	Frequency	Rate (%)	Cumulative Frequency	Property	Flavor	Meridian Tropism
1	Astragali Radix(Huangqi)	184	7.07%	7.07%	warm	sweet	spleen, lung
2	Atractylodis Macrocephalae Rhizoma(Baizhu)	129	4.96%	12.03%	warm	Sweet, bitter	spleen, stomach
3	Ligustri Lucidi Fructus(Nvzhenzi)	125	4.80%	16.83%	cool	Sweet, bitter	liver, kidney
4	Angelicae Sinensis Radix(Danggui)	120	4.61%	21.44%	warm	Sweet, pungent	liver, heart, spleen
5	Lycii Frucyus(Gouqizi)	119	4.57%	26.02%	mild	Sweet	liver, kidney
6	Codonopsis Radix (Dangshen)	101	3.88%	29.90%	mild	Sweet	spleen, lung
7	Poria(Fuling)	99	3.80%	33.70%	mild	Sweet, tasteless	heart, lung, spleen, kidney
8	Rehmanniae Radix Praepapata(Shudihuang)	97	3.73%	37.43%	warm	Sweet	liver, kidney
9	Spatholobi Caulis(Jixueteng)	96	3.69%	41.12%	warm	Bitter, Sweet	liver, kidney
10	Glycyrrhizae Radix Et Rhizoma(Gancao)	90	3.46%	44.58%	mild	Sweet	heart, lung, spleen, stomach
11	Psoraleae Fructus(Buguzhi)	80	3.07%	47.65%	warm	Pungent, bitter	kidney, spleen
12	Corni Frucyus(Shanzhuyu)	55	2.11%	49.77%	warm	Sour, astringent	liver, kidney
13	Aisni Corii Colla(Ejiao)	50	1.92%	51.69%	mild	Sweet	lung, liver, kidney
14	Cuscutae Semen(Tusizi)	50	1.92%	53.61%	mild	Pungent, Sweet	liver, kidney, spleen
15	Citri Reticulatae Pericarpium(Chenpi)	47	1.81%	55.42%	warm	Bitter, pungent	spleen, lung
16	Polygoni Multiflori Radix(Heshouwu)	45	1.73%	57.15%	warm	Bitter, Sweet, astringent	liver, heart, kidney
17	Polygonati Rhizoma(Huangjing)	45	1.73%	58.88%	mild	Sweet	spleen, lung, kidney
18	Dioscoreae Rhizoma(Shanyao)	45	1.73%	60.61%	mild	Sweet	spleen, lung, kidney
19	Epimedii Folium(Yinyanghuo)	45	1.73%	62.34%	warm	Pungent, Sweet	liver, kidney
20	Pseudostellariae Radix(Taizishen)	37	1.42%	63.76%	mild	Sweet, bitter	spleen, lung
21	Paeoniae Radix Alba(Baishao)	35	1.35%	65.10%	cold	Bitter, sour	liver, spleen
22	Jujubae Fructus(Dazao)	33	1.27%	66.37%	warm	Sweet	spleen, stomach, heart
23	Antler glue (Lujiaojiao)	30	1.15%	67.52%	warm	Sweet, salty	kidney, liver
24	Pinelliae Rhizoma(Banxia)	29	1.11%	68.64%	warm	pungent	spleen, stomach, lung
25	Ginseng Radix Et Rhizoma(Renshen)	26	1.00%	69.64%	warm	Sweet, bitter	spleen, lung, heart, kidney
26	Crataegi Fructus(Shanzha)	25	0.96%	70.60%	warm	Sour, Sweet	spleen, stomach, liver
27	Amomi Fructus(Sharen)	24	0.92%	71.52%	warm	pungent	spleen, stomach, kidney
28	Rehmanniae Radix(Shengdihuang)	21	0.81%	72.33%	cold	Sweet	heart, liver, kidney
29	Coicis Semen(Yiyiren)	21	0.81%	73.13%	cool	Sweet, tasteless	spleen, stomach, lung
30	Eucommiae Cortex(Duzhong)	20	0.77%	73.90%	warm	Sweet	liver, kidney
31	Ecliptae Herba(Mohanlian)	20	0.77%	74.67%	cold	Sweet, sour	kidney, liver
32	Gigerii Endothelium Corneum(Jineijin)	18	0.69%	75.36%	mild	Sweet	spleen, stomach, small intestine, urinary bladder

**Table 2:** Properties of herbs.

Property	Quantity	Categories (%)	Frequency of Occurrence in Formulae(%)^*＊*^
warm	17	7 (53.85)	1085 (41.70)
mild	10	3 (23.08)	654 (25.13)
cold	3	2 (15.38)	76 (2.92)
cool	2	2 (15.38)	146 (5.61)

tip: *＊*a single Chinese herb may involve multiple properties, so it is repeated to count the herb quantity and frequency.

**Table 3:** Five flavors of herbs.

Flavors	Quantity	Categories (%)	Frequency of Occurrence in Formulae (%)^*＊*^
sweet	26	5 (38.46)	169 (64.99)
bitter	9	3 (23.08)	620 (23.83)
pungent	7	4 (30.77)	395 (15.18)
sour	4	3 (23.08)	135 (4.42)
salty	1	1 (7.70)	30 (1.15)

tip: ^*＊*^a single Chinese herb may involve multiple tastes, so it is repeated to count the herb quantity and frequency.

**Table 4:** Meridian tropism of herbs.

Meridian Tropism	Quantity	Categories (%)	Frequency of Occurrence in Formulae (%)^*＊*^
spleen	20	6 (46.15)	1238 (47.58)
kidney	19	6 (46.15)	1092 (41.97)
liver	16	5 (38.46)	953 (36.63)
lung	12	4 (30.77)	774 (29.75)
heart	7	3 (23.08)	434 (16.68)
stomach	9	5 (38.46)	369 (14.18)
urinary bladder	1	1 (7.69)	18 (0.69)
small intestine	1	1 (7.69)	18 (0.69)

tip: ^*＊*^a single Chinese herb may involve multiple meridian tropisms, so it is repeated to count the herb quantity and frequency.

**Table 5:** Frequency of herb categories.

No.	Herb Category (quantity)	Frequency	Rate(%)	Cumulative Frequency(%)
1	deficiency-tonifying herbs (37)	1681	64.6	64.6
2	blood-activating and stasis-resolving herbs (6)	147	5.64	70.24
3	dampness-draining diuretic herbs (5)	139	5.34	75.58
4	digestant herbs (5)	78	3	78.58
5	heat-clearing herbs (5)	72	2.77	81.35
6	Astringent herbs (2)	66	2.54	83.89
7	qi-regulating herbs (2)	63	2.42	86.31
8	dampness-resolving medicina (2)	29	1.11	87.42
9	cough-suppressing and panting-calming herbs (1)	29	1.11	88.53
10	interior-warming herbs (2)	27	1.04	89.57
11	Exterior-releasing herbs (2)	13	0.5	90.07
12	Hemostatic herbs (1)	13	0.5	90.57
13	Nerve-soothing herbs (1)	5	0.19	90.76

**Table 6:** Association rules of herbs in BMS formulae.

No.	Herbal Suits	Support (%)	Confidence (%)	Lift
1	{Atractylodis Macrocephalae Rhizoma(Baizhu)} => {Astragali Radix(Huangqi)}	47.06	86.82	1.12
2	{Angelicae Sinensis Radix(Danggui)} => {Astragali Radix(Huangqi)}	44.54	88.33	1.16
3	{Codonopsis Radix (Dangshen)} => {Astragali Radix(Huangqi)}	36.13	86	1.13
4	{Spatholobi Caulis(Jixueteng)} => {Astragali Radix(Huangqi)}	35.29	87.5	1.14
5	{Atractylodis Macrocephalae Rhizoma(Baizhu), Ligustri Lucidi Fructus(Nvzhenzi)} => {Astragali Radix(Huangqi)}	29.83	91.03	1.19
6	{Atractylodis Macrocephalae Rhizoma(Baizhu), Codonopsis Radix (Dangshen)} => {Astragali Radix(Huangqi)}	29.41	87.5	1.14
7	{Atractylodis Macrocephalae Rhizoma(Baizhu), Poria(Fuling)} => {Astragali Radix(Huangqi)}	29	86.25	1.13
8	{Poria(Fuling), Astragali Radix(Huangqi)} => {Atractylodis Macrocephalae Rhizoma(Baizhu)}	28.99	86.25	1.59
9	{Atractylodis Macrocephalae Rhizoma(Baizhu), Angelicae Sinensis Radix(Danggui)} => {Astragali Radix(Huangqi)}	27.73	89.19	1.17
10	{Atractylodis Macrocephalae Rhizoma(Baizhu), Glycyrrhizae Radix Et Rhizoma(Gancao)} => {Astragali Radix(Huangqi)}	23.95	85.07	1.11
11	{Atractylodis Macrocephalae Rhizoma(Baizhu), Lycii Frucyus(Gouqizi)} => {Astragali Radix(Huangqi)}	23.95	85.07	1.11
12	{Poria(Fuling), Ligustri Lucidi Fructus(Nvzhenzi)} => {Astragali Radix(Huangqi)}	22.69	87.1	1.14
13	{Spatholobi Caulis(Jixueteng), Ligustri Lucidi Fructus(Nvzhenzi)} => {Astragali Radix(Huangqi)}	22.29	86.88	1.14
14	{Angelicae Sinensis Radix(Danggui), Codonopsis Radix (Dangshen)} => {Astragali Radix(Huangqi)}	22.27	91.38	1.19
15	{Atractylodis Macrocephalae Rhizoma(Baizhu), Spatholobi Caulis(Jixueteng)} => {Astragali Radix(Huangqi)}	21.43	94.44	1.24
16	{Codonopsis Radix (Dangshen), Poria(Fuling)} => {Atractylodis Macrocephalae Rhizoma(Baizhu)}	21.43	91.07	1.68
17	{Angelicae Sinensis Radix(Danggui), Codonopsis Radix (Dangshen)} => {Atractylodis Macrocephalae Rhizoma(Baizhu)}	21.43	87.93	1.62
18	{Angelicae Sinensis Radix(Danggui), Ligustri Lucidi Fructus(Nvzhenzi)} => {Astragali Radix(Huangqi)}	21.43	92.72	1.21
19	{Angelicae Sinensis Radix(Danggui), Spatholobi Caulis(Jixueteng)} => {Astragali Radix(Huangqi)}	21.01	90.91	1.19
20	{Poria(Fuling), Glycyrrhizae Radix Et Rhizoma(Gancao)} => {Atractylodis Macrocephalae Rhizoma(Baizhu)}	21.01	87.72	1.62
21	{Glycyrrhizae Radix Et Rhizoma(Gancao), Ligustri Lucidi Fructus(Nvzhenzi)} => {Astragali Radix(Huangqi)}	21.01	92.59	1.21
22	{Codonopsis Radix (Dangshen), Poria(Fuling)} => {Astragali Radix(Huangqi)}	21.01	89.29	1.17
23	{Codonopsis Radix (Dangshen), Ligustri Lucidi Fructus(Nvzhenzi)} => {Astragali Radix(Huangqi)}	21.01	86.21	1.13
24	{Angelicae Sinensis Radix(Danggui), Poria(Fuling)} => {Astragali Radix(Huangqi)}	20.17	85.71	1.12
25	{Atractylodis Macrocephalae Rhizoma(Baizhu), Poria(Fuling), Ligustri Lucidi Fructus(Nvzhenzi)} => {Astragali Radix(Huangqi)}	20.17	92.31	1.21
26	{Poria(Fuling), Astragali Radix(Huangqi, Ligustri Lucidi Fructus(Nvzhenzi)} => {Atractylodis Macrocephalae Rhizoma(Baizhu)}	20.17	88.89	1.64
